# Supplementary material for: Dual Geometry Schemes in Tetrel Bonds: Complexes between TF4 (T = Si, Ge, Sn) and Pyridine Derivatives
Source: Molecules. 2019 Jan 21;24(2):376. doi: 10.3390/molecules24020376 (PMC6359171; doi:10.3390/molecules24020376)
Supplement: Supplementary file 1 [file molecules-24-00376-s001.pdf]

Supplementary Information

Dual Geometry Schemes in Tetrel Bonds: Complexes between TF_4 (T = Si, Ge, Sn) and Pyridine Derivatives

Wiktor Zierkiewicz [1,*], Mariusz Michalczyk [1], Rafał Wysokiński [1] and Steve Scheiner [2,*]

1 Faculty of Chemistry, Wrocław University of Science and Technology, Wybrzeże Wyspiańskiego 27, 50-370 Wrocław, Poland; mariusz.michalczyk@pwr.edu.pl (M.M.); rafal.wysokinski@pwr.edu.pl (R.W.)
2 Department of Chemistry and Biochemistry, Utah State University, Logan, 84322-0300 UT, USA
* Correspondence: wiktor.zierkiewicz@pwr.edu.pl (W.Z.); steve.scheiner@usu.edu (S.S.)

axial

pyridine···SiF_4

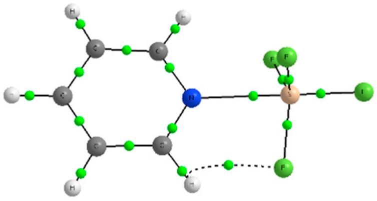

pyridine···GeF_4

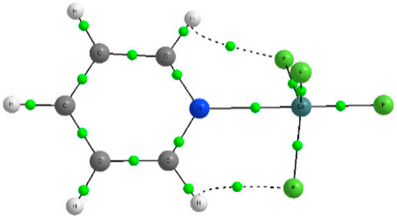

pyridine···SnF_4

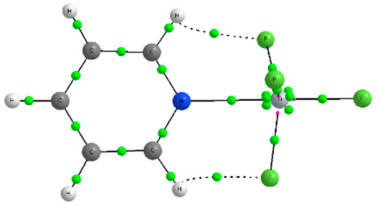

4-pyridinol···SiF_4

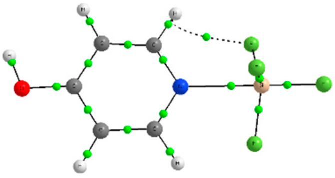

4-pyridinol···GeF_4

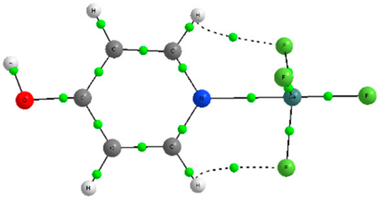

4-pyridinol···SnF_4

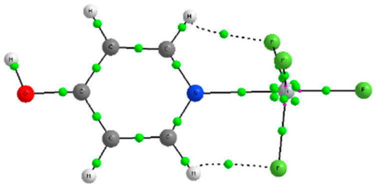

4-methyl-pyridine···SiF_4

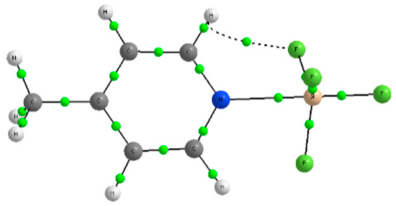

4-methyl-pyridine···GeF_4

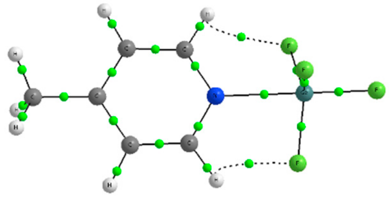

4-methyl-pyridine···SnF_4

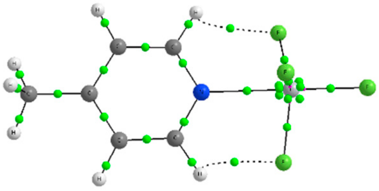

4-trifluoromethane-pyridine···SiF_4

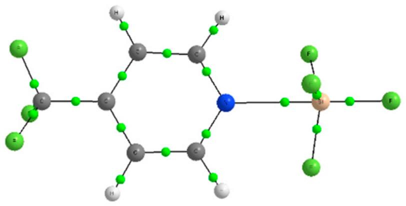

4-trifluoromethane-pyridine···GeF_4

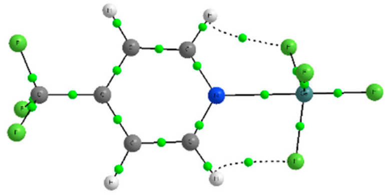

4-trifluoromethane-pyridine···SnF_4

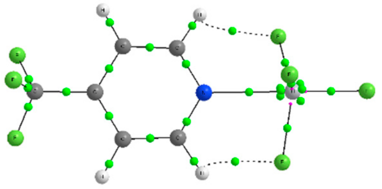

equatorial

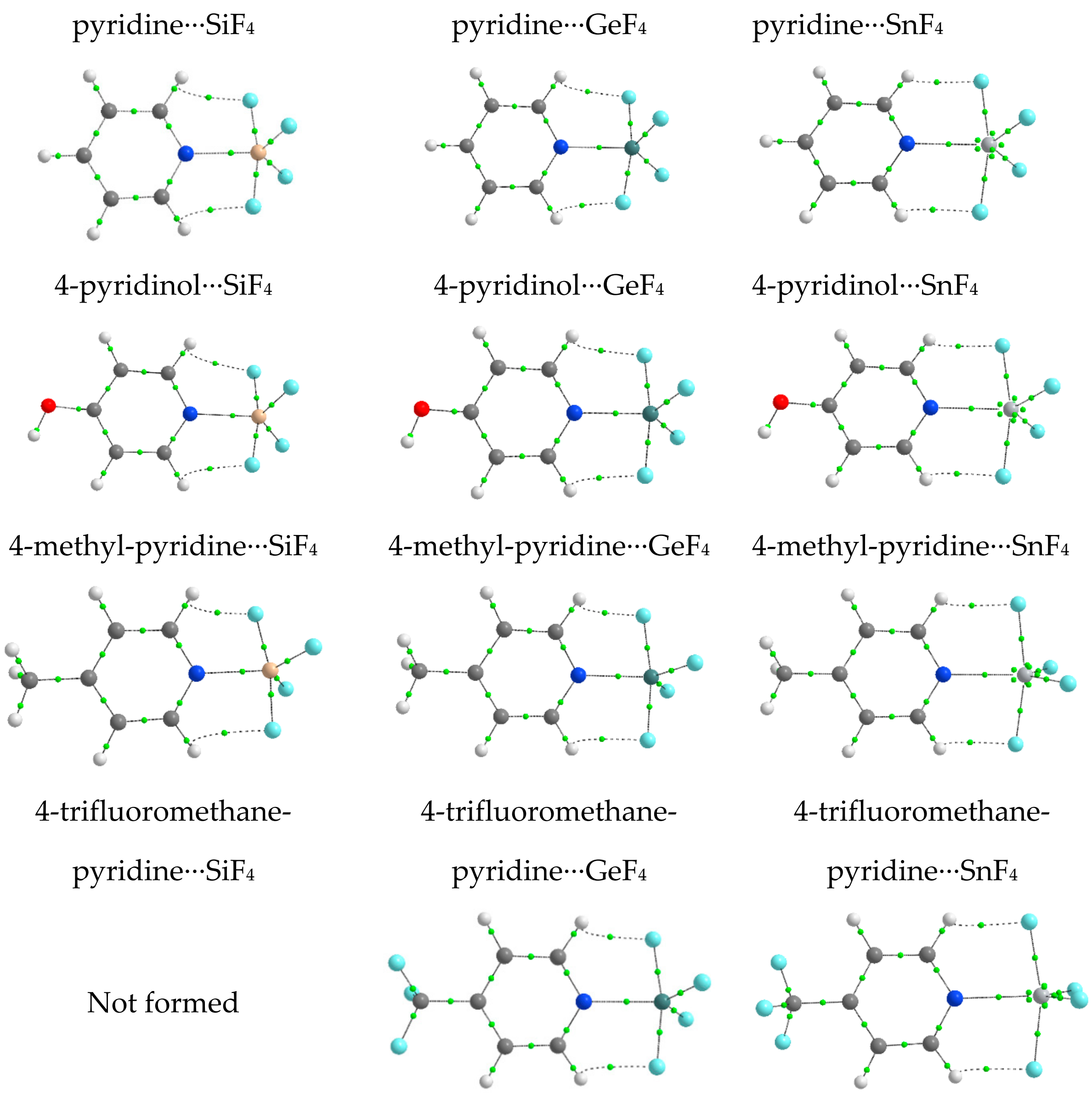

Fig. S1. AIM molecular graphs of axial and equatorial TF_4 (T = Si, Ge, Sn) complexes with pyridine derivatives at the MP2/cc-pVTZ level. Small green dots represent bond critical points.

Table S1. The delocalization index (DI) of T···N interaction in TF_4 complexes with pyridine and its derivatives. Data obtained at the MP2/cc-pVTZ level of theory.[a]

Axial		Equatorial	
Complex	DI	Complex	DI
Py···SiF_4	0.101	Py···SiF_4	0.170
Py···GeF_4	0.248	Py···GeF_4	0.336

4-HOPy···SiF_4	0.106	4-HOPy···SiF_4	0.174
4-HOPy···GeF_4	0.256	4-HOPy···GeF_4	0.344
4-MePy···SiF_4	0.104	4-MePy···SiF_4	0.173
4-MePy···GeF_4	0.254	4-MePy···GeF_4	0.340
4-TFMPy···SiF_4	0.088	4-TFMPy···SiF_4	-
4-TFMPy···GeF_4	0.234	4-TFMPy···GeF_4	0.323

[a] values for Sn complexes are not available in AIM analysis due to the basis set used during investigation.

Table S2. Gibbs free energies of axial and equatorial conformers as well as TS for TF_4 (T: Si, Ge, Sn) complexes with Py, calculated at the MP2/cc-pVTZ level of theory.

	axial	TS	equatorial
Py···SiF_4	0.00	11.79 (-19)[a]	10.14
Py···GeF_4	0.00	8.38 (-36)	6.79
Py···SnF_4	0.00	1.46 (-18)	3.46

[a] imaginary frequency of TS is given in parenthesis.

Table S3. Secondary minima for TF_4 complexes with pyridine and its derivatives. Data obtained at the MP2/cc-pVTZ level of theory. E_{int} corrected for BSSE (in kcal/mol). Parameters: distances in Å, angles in degrees.

System	E_{int}	Parameters	Structure
Py···SiF_4	-1.50	R(Si···C3): 3.590 F1-Si···C3: 175.7	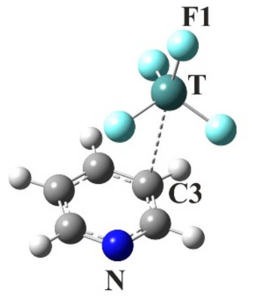
Py···GeF_4	-1.86	R(Ge···C3): 3.452 F1-Ge···C3: 175.7	
Py···SnF_4	-5.47	R(Sn···C3): 2.955 F1-Sn···C3:	

		175.7	
Py···SiF_4	-1.46	R(Si···C4): 3.599 F1-Si···C4: 175.1	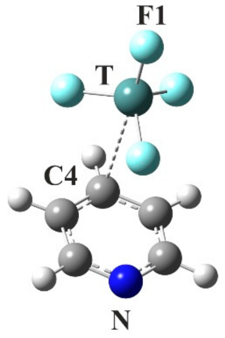
Py···GeF_4	-1.81	R(Ge···C4): 3.356 F1-Ge···C4: 175.2	
Py···SnF_4	-4.85	R(Sn···C4): 3.008 F1-Sn···C4: 175.1	
4-HPy···SiF_4	-2.58	R(Si···O): 2.991 F1-Si···O: 177.8	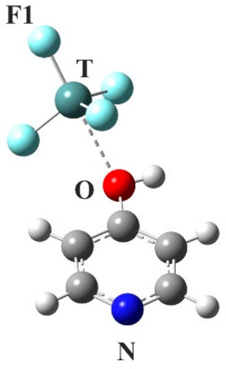
4-HPy···GeF_4	-6.36	R(Ge···O): 2.531 F1-Ge···O: 176.6	
4-HPy···SnF_4	-17.02	R(Sn···O): 2.303 F1-Sn···O: 173.8	
4-HPy···SiF_4	-1.88	R(Si···C3): 3.488 F1-Si···C3: 173.3	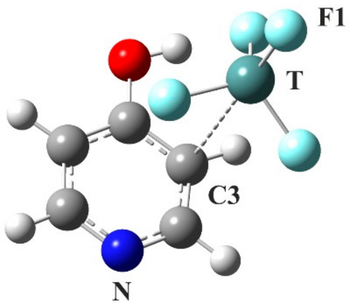
4-HPy···GeF_4	-2.49	R(Ge···C3): 3.350 F1-Ge···C3: 174.0	

4-HPy···SnF_4	-12.48	R(Sn···C3): 2.664<br>F1-Sn···C3:<br>174.7	
4-HPy···SiF_4	-1.68	R(Si···C4): 3.542<br>F1-Si···C4:<br>172.3	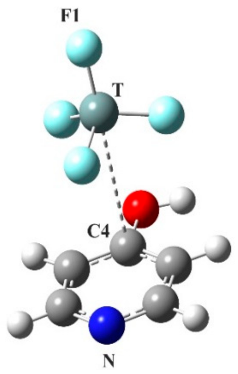
4-HPy···GeF_4	-2.08	R(Ge···C4): 3.474<br>F1-Ge···C4:<br>171.9	
4-HPy···SnF_4	-5.04	R(Sn···C4): 3.190<br>F1-Sn···C4:<br>172.3	
4-HPy···SiF_4	-	-	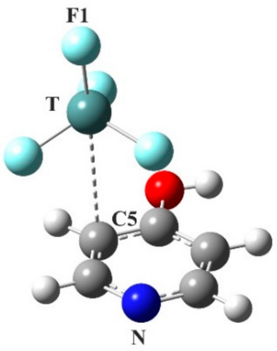
4-HPy···GeF_4	-1.78	R(Ge···C5): 3.461<br>F1-Ge···C5:<br>174.9	
4-HPy···SnF_4	-5.77	R(Sn···C5): 2.958<br>F1-Sn···C5:<br>176.6	
4-MPy···SiF_4	-1.89	R(Si···C4): 3.579<br>F1-Si···C4:<br>174.0	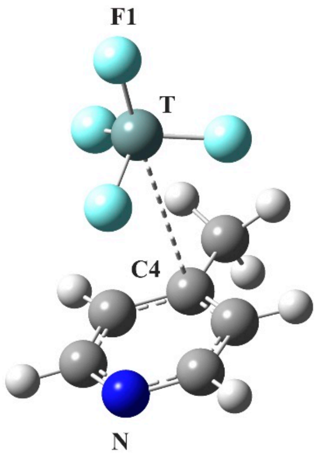
4-MPy···GeF_4	-2.30	R(Ge···C4): 3.507<br>F1-Ge···C4:<br>173.2	
4-MPy···SnF_4		R(Sn···C4):<br>F1-Sn···C4:	

4-MPy···SiF_4	-1.88	R(Si···C5): 3.591 F1-Si···C5: 174.6	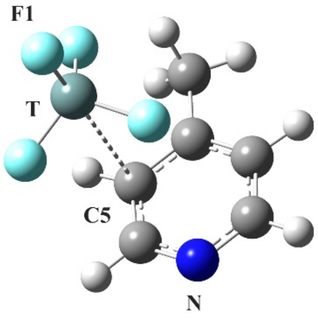
4-MPy···GeF_4	-2.30	R(Ge···C5): 3.441 F1-Ge···C5: 175.3	
4-MPy···SnF_4	-7.14	R(Sn···C5): 2.919 F1-Sn···C5: 176.3	
4-TFMPy···SiF_4	-1.40	R(Si···C5): 3.592 F1-Si···C5: 173.7	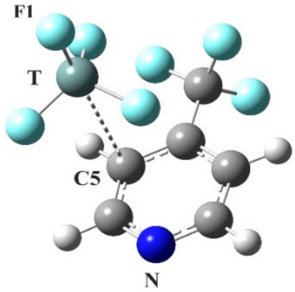
4-TFMPy···GeF_4	-1.97	R(Ge···C5): 3.520 F1-Ge···C5: 172.5	
4-TFMPy···SnF_4	-2.52	R(Sn···C5): 3.329 F1-Sn···C5: 172.6	

Table S4. Interaction energies (E_{int}, kcal mol^{-1}) corrected for BSSE calculated at the MP2/cc-pVTZ (I), BLYP-D3/Def2TZVPP (II) and B2PLYP/def2TZVPP (III) levels of theory.

	axial complexes			equatorial complexes		
	(I)	(II)	(III)	(I)	(II)	(III)
Py···SiF_4	-26.75	-22.50	-25.72	-50.09	-47.38	-50.79
Py···GeF_4	-34.73	-34.37	-35.53	-52.02	-48.71	-52.99
Py···SnF_4	-39.68	-37.87	-39.61	-50.66	-44.97	-49.92

Table S5. Molecular coordinates for minima of primary TF_4 complexes with pyridine and its derivatives. Data obtained at the MP2/cc-pVTZ level of theory.

System	axial				equatorial			
Py···SiF_4	F	1.583251	0.030612	1.581591	N	0.478657	0.003751	-0.001309
	F	1.733921	1.364867	-0.814763	C	1.159679	-1.148107	0.150653
	F	3.498622	-0.001105	0.102934	C	2.545227	-1.173893	0.172045
	F	1.732174	-1.397781	-0.762173	C	3.252948	0.009130	-0.002627
	C	-0.937672	-1.153323	-0.067588	C	2.540484	1.189400	-0.176595
	C	-2.324130	-1.186764	-0.010080	C	1.155059	1.158236	-0.153877
	C	-2.314874	1.201157	-0.010084	H	0.560191	-2.037892	0.243246
	C	-0.928870	1.156823	-0.067700	H	3.050773	-2.116717	0.314990
	N	-0.249414	-0.000918	-0.098756	H	3.042230	2.134185	-0.319976
	H	-0.347562	-2.055488	-0.097683	H	0.552045	2.045705	-0.245774
	H	-2.831506	-2.139621	0.009887	Si	-1.494256	-0.000661	0.000378
	H	-2.814850	2.157915	0.009915	F	-1.315694	1.470473	0.722991
	H	-0.332029	2.054646	-0.098166	F	-1.310272	-1.469667	-0.725232
	Si	1.890801	-0.001351	0.009801	F	-2.267353	-0.626411	1.261278
	C	-3.030559	0.009900	0.018751	F	-2.280857	0.619448	-1.255073
	H	-4.110333	0.014085	0.062396	H	4.333575	0.011223	-0.003142
Py···GeF_4	F	1.561493	0.036062	1.699323	N	0.504357	0.003763	-0.001411
	F	1.730588	1.470523	-0.878121	C	1.181000	-1.155150	0.115007
	F	3.577296	-0.000222	0.107835	C	2.566601	-1.179762	0.130886
	F	1.730190	-1.508235	-0.817141	C	3.273374	0.009235	-0.002702
	C	-0.940411	-1.158256	-0.068688	C	2.561783	1.195428	-0.135604
	C	-2.326211	-1.187611	-0.010134	C	1.176301	1.165339	-0.118424
	C	-2.316228	1.201995	-0.008664	H	0.577205	-2.044418	0.181145

	C	-0.930903	1.160929	-0.067388	H	3.072871	-2.126713	0.239870
	N	-0.258530	-0.001491	-0.100621	H	3.064204	2.144378	-0.245019
	H	-0.346476	-2.057332	-0.101370	H	0.568936	2.052219	-0.183892
	H	-2.834531	-2.139791	0.009938	Ge	-1.519862	-0.000760	0.000512
	H	-2.816565	2.158365	0.012623	F	-1.355628	1.631132	0.631125
	H	-0.329929	2.055447	-0.099529	F	-1.349442	-1.630803	-0.633275
	Ge	1.858550	-0.001373	0.005787	F	-2.329595	-0.552378	1.408138
	C	-3.030842	0.010079	0.019885	F	-2.343670	0.545321	-1.401169
	H	-4.110521	0.014563	0.064547	H	4.353999	0.011371	-0.003208
Py···SnF_4	F	1.632243	0.007719	1.900168	N	0.568934	0.004530	-0.001195
	F	1.755635	1.678662	-0.896749	C	1.244386	-1.160434	-0.079562
	F	3.843686	-0.007766	0.043847	C	2.630681	-1.188931	-0.080701
	F	1.746950	-1.693981	-0.878244	C	3.340172	0.003301	0.000316
	C	-0.980802	-1.158875	-0.064695	C	2.631637	1.196151	0.080551
	C	-2.366531	-1.188104	-0.009038	C	1.245317	1.168869	0.077899
	C	-2.360076	1.204063	-0.021107	H	0.634302	-2.047067	-0.139871
	C	-0.974528	1.166787	-0.076437	H	3.135068	-2.141104	-0.144817
	N	-0.303472	0.001995	-0.097142	H	3.136782	2.147885	0.145216
	H	-0.382520	-2.055898	-0.099119	H	0.635930	2.056027	0.137550
	H	-2.874919	-2.140145	0.013062	Sn	-1.649221	0.005160	-0.002623
	H	-2.863308	2.159013	-0.008623	F	-1.423842	1.923829	0.128876
	H	-0.371433	2.060184	-0.119948	F	-1.424977	-1.913724	-0.132784
	Sn	1.938245	-0.003205	0.011146	F	-2.564168	-0.108391	1.665273
	C	-3.071206	0.010038	0.014141	F	-2.559433	0.119270	-1.673068
	H	-4.150992	0.013163	0.057019	H	4.420870	0.002830	0.000917
4-HPy···SiF_4	F	1.579004	-0.056234	1.586251	N	0.173409	0.004115	-0.001711
	F	1.722027	1.417468	-0.734689	C	0.864521	-1.148455	0.086046
	F	3.482915	0.003977	0.099114	C	2.244948	-1.180292	0.106036

	F	1.725270	-1.353310	-0.833681	C	2.956249	0.013029	0.001991
	Si	1.872885	0.003499	0.011075	C	2.239373	1.202869	-0.103696
	C	-0.930663	-1.151900	-0.068551	C	0.861126	1.162531	-0.087200
	C	-2.310571	-1.205760	-0.012256	H	0.274105	-2.047474	0.132209
	C	-2.324416	1.185084	-0.013314	H	2.749613	-2.132315	0.193012
	C	-0.941252	1.149790	-0.069418	O	4.300275	0.085664	-0.002792
	N	-0.244974	0.004063	-0.100359	H	2.757064	2.145775	-0.188186
	H	-0.338758	-2.052956	-0.097889	H	0.264815	2.057434	-0.135004
	H	-2.825528	-2.154062	0.008346	H	4.669920	-0.801812	0.076615
	H	-2.833583	2.138984	0.005625	Si	-1.787012	-0.003669	-0.000696
	H	-0.362305	2.059286	-0.098231	F	-1.628062	1.488440	0.685969
	C	-3.030998	-0.014297	0.015970	F	-1.617162	-1.494792	-0.689397
	O	-4.378582	-0.091063	0.069543	F	-2.557952	-0.599566	1.277558
	H	-4.745161	0.800552	0.085744	F	-2.571424	0.582651	-1.274941
4-HPy···GeF_4	F	1.272295	-0.122939	1.703185	N	0.198627	0.004696	-0.000294
	F	1.429758	1.574627	-0.712722	C	0.884925	-1.153788	0.041404
	F	3.279666	0.020987	0.110218	C	2.265085	-1.184682	0.056865
	F	1.448529	-1.406479	-0.952914	C	2.976044	0.012813	0.006911
	Ge	1.559297	0.016312	0.012381	C	2.260127	1.207211	-0.046462
	C	-1.218323	-1.150853	-0.140154	C	0.882045	1.168626	-0.038036
	C	-2.597152	-1.207975	-0.087580	H	0.289198	-2.050635	0.052134
	C	-2.617506	1.181101	0.052199	H	2.770220	-2.139393	0.100958
	C	-1.235521	1.155831	-0.005120	O	4.318913	0.085712	0.007000
	N	-0.542184	0.011325	-0.104308	H	2.778961	2.152412	-0.087303
	H	-0.619866	-2.043672	-0.223582	H	0.280989	2.061718	-0.051974
	H	-3.110503	-2.156602	-0.122743	H	4.690031	-0.803941	0.047079
	H	-3.129617	2.130447	0.127181	Ge	-1.813353	-0.003271	-0.002547
	H	-0.653552	2.063057	0.017407	F	-1.672295	1.647427	0.587596

	C	-3.319913	-0.021039	0.010642	F	-1.657229	-1.653042	-0.594961
	O	-4.665212	-0.105124	0.058874	F	-2.623655	-0.521179	1.418840
	H	-5.037417	0.781826	0.128068	F	-2.634825	0.503450	-1.421395
4-HPy···SnF_4	F	1.102260	-0.089996	1.898910	N	0.258935	0.005147	0.005395
	F	1.191668	1.751880	-0.781977	C	0.945113	-1.149054	-0.115828
	F	3.293359	0.023758	0.023580	C	2.326051	-1.180476	-0.110912
	F	1.215159	-1.631136	-0.972786	C	3.036133	0.011429	0.022885
	Sn	1.386924	0.011248	0.008711	C	2.320755	1.201199	0.147825
	C	-1.521137	-1.158795	-0.146788	C	0.941977	1.163867	0.135400
	C	-2.899960	-1.207783	-0.094177	H	0.345099	-2.039164	-0.216108
	C	-2.909475	1.1838400	0.050602	H	2.832472	-2.130392	-0.211055
	C	-1.528146	1.154061	-0.006799	O	4.379007	0.083273	0.038838
	N	-0.842235	0.003131	-0.103800	H	2.840652	2.140947	0.253088
	H	-0.923478	-2.052026	-0.239608	H	0.336588	2.050987	0.228079
	H	-3.418941	-2.153147	-0.133872	H	4.750521	-0.802189	-0.055081
	H	-3.418364	2.134926	0.124703	Sn	-1.944357	-0.002163	-0.007800
	H	-0.939360	2.057952	0.008906	F	-1.733013	1.912457	0.198634
	C	-3.616105	-0.016561	0.008238	F	-1.715911	-1.916003	-0.214498
	O	-4.960346	-0.095113	0.057458	F	-2.868720	-0.186973	1.649192
	H	-5.330511	0.792653	0.129261	F	-2.857495	0.171242	-1.672240
4-MPy···SiF_4	F	1.596191	-0.049713	1.586048	N	0.153965	-0.000715	-0.002556
	F	1.750016	1.407929	-0.744518	C	0.838609	1.154507	-0.104617
	F	3.505578	-0.005706	0.105168	C	2.222416	1.179400	-0.127821
	F	1.742787	-1.358318	-0.827835	C	2.953388	-0.001452	-0.009023
	Si	1.896290	0.000359	0.012056	C	2.222445	-1.182092	0.110282
	C	-0.912769	-1.147049	-0.058468	C	0.838404	-1.156354	0.094303
	C	-2.297466	-1.186050	-0.000799	H	0.242546	2.049679	-0.159514
	C	-2.305702	1.186625	-0.016569	H	2.723550	2.131122	-0.233555

	C	-0.920402	1.156724	-0.073566	H	2.723579	-2.135088	0.203449
	N	-0.228048	0.007062	-0.098120	H	0.242162	-2.051572	0.146325
	H	-0.321233	-2.048806	-0.079420	Si	-1.811461	0.000364	0.000909
	H	-2.795713	-2.145095	0.030515	F	-1.641428	-1.456068	-0.754976
	H	-2.810574	2.142525	0.001961	F	-1.636363	1.456322	0.756682
	H	-0.334669	2.061711	-0.105914	F	-2.591826	0.650972	-1.243975
	C	-3.032784	-0.001909	0.019522	F	-2.589182	-0.648724	1.248282
	C	-4.530966	-0.007239	0.050072	C	4.450569	0.001936	0.017088
	H	-4.909052	-0.884631	0.570157	H	4.851780	-0.935023	-0.362088
	H	-4.916087	0.882970	0.542381	H	4.850830	0.820782	-0.576387
	H	-4.928885	-0.024974	-0.964998	H	4.806240	0.127607	1.040276
4-MPy···GeF_4	F	1.284625	-0.067539	1.707071	N	0.488673	-0.001601	-0.000735
	F	1.448863	1.548612	-0.764714	C	1.168823	1.158422	-0.078765
	F	3.298429	0.023607	0.119271	C	2.552644	1.181610	-0.100312
	F	1.468455	-1.431290	-0.911338	C	3.282854	-0.002680	-0.014584
	Ge	1.578742	0.017530	0.013942	C	2.552608	-1.186650	0.075393
	C	-1.202789	-1.151419	-0.126292	C	1.168655	-1.162299	0.068405
	C	-2.586900	-1.193893	-0.072119	H	0.568380	2.051726	-0.113379
	C	-2.601961	1.177589	0.047322	H	3.054431	2.135378	-0.181231
	C	-1.217476	1.158313	-0.009975	H	3.054386	-2.141602	0.140898
	N	-0.528160	0.009398	-0.100372	H	0.567834	-2.055332	0.103227
	H	-0.604251	-2.045226	-0.199330	Ge	-1.528377	0.000529	0.006303
	H	-3.082942	-2.154104	-0.095642	F	-1.375212	-1.627768	-0.638940
	H	-3.110159	2.128934	0.120084	F	-1.366371	1.626350	0.655821
	H	-0.630374	2.062465	0.007492	F	-2.341559	0.564642	-1.395261
	C	-3.324511	-0.014177	0.014975	F	-2.349169	-0.558025	1.405829
	C	-4.822254	-0.025129	0.044490	C	4.779828	-0.001036	0.007501
	H	-5.198376	-0.936293	0.504157	H	5.179290	-0.919170	-0.416890

	H	-5.209477	0.829549	0.594560	H	5.179169	0.845201	-0.546645
	H	-5.218599	0.024341	-0.970160	H	5.137479	0.074623	1.034939
4-MPy···SnF_4	F	1.099674	-0.092257	1.897898	N	0.825201	0.001440	-0.002223
	F	1.218760	1.744258	-0.794690	C	1.507559	-1.149636	-0.169876
	F	3.308872	0.015206	0.046342	C	2.892037	-1.168451	-0.195977
	F	1.230695	-1.629130	-0.975337	C	3.620376	0.011031	-0.047664
	Sn	1.402741	0.010238	0.010511	C	2.889129	1.185873	0.121601
	C	-1.506883	-1.152652	-0.145581	C	1.504700	1.157445	0.141336
	C	-2.891216	-1.189014	-0.097187	H	0.903845	-2.035700	-0.282031
	C	-2.899236	1.184518	0.031365	H	3.395594	-2.114355	-0.337233
	C	-1.514717	1.162923	-0.020186	H	3.390325	2.136626	0.236180
	N	-0.831371	0.008690	-0.106216	H	0.898816	2.040093	0.267796
	H	-0.908090	-2.045807	-0.233463	Sn	-1.384549	-0.005989	0.032648
	H	-3.391721	-2.146210	-0.138817	F	-1.168342	1.902142	0.286519
	H	-3.406198	2.137214	0.093183	F	-1.163556	-1.912547	-0.228788
	H	-0.922027	2.064386	-0.010957	F	-2.271339	-0.233014	1.704945
	C	-3.623879	-0.006295	-0.003742	F	-2.328438	0.214612	-1.608954
	C	-5.119295	-0.015941	0.081392	C	5.117534	0.012186	-0.042771
	H	-5.434209	-0.072979	1.123838	H	5.487077	-0.125155	0.974022
	H	-5.542191	0.891040	-0.344498	H	5.512287	-0.797489	-0.652080
	H	-5.536210	-0.874927	-0.439335	H	5.510182	0.955737	-0.414519
4-TFMPy···SiF_4	F	2.538870	0.042463	1.574390	-			
	F	2.678089	1.356864	-0.819472				
	F	4.468055	0.003717	0.095524				
	F	2.678848	-1.395762	-0.747916				
	Si	2.864569	0.001253	0.010075				
	C	-0.005884	-1.155168	-0.042720				

	C -1.391526 -1.196340 0.016468<br>C -1.389908 1.199076 -0.029632<br>C -0.004398 1.153793 -0.087181<br>N 0.678150 -0.001763 -0.096301<br>H 0.585893 -2.056919 -0.054150<br>H -1.903903 -2.145134 0.062284<br>H -1.901060 2.149595 -0.020347<br>H 0.588484 2.053747 -0.133357<br>C -2.094096 0.002428 0.025451<br>C -3.597297 0.003858 0.040967<br>F -4.079550 1.096778 0.641697<br>F -4.083793 -0.018099 -1.207695<br>F -4.081350 -1.065936 0.680570	
4-TFMPy···GeF_4	F 2.176379 -0.053959 1.699925<br>F 2.348644 1.535828 -0.776824<br>F 4.211231 0.014772 0.119543<br>F 2.354308 -1.435259 -0.898260<br>Ge 2.495844 0.015766 0.013677<br>C -0.317914 -1.144759 -0.116256<br>C -1.702803 -1.188662 -0.062687<br>C -1.707425 1.206399 0.034906<br>C -0.322381 1.172333 -0.021840<br>N 0.356533 0.016356 -0.100045<br>H 0.280439 -2.039173 -0.181826<br>H -2.213238 -2.139438 -0.074588<br>H -2.221522 2.153017 0.100319<br>H 0.272512 2.071413 -0.014330<br>C -2.407420 0.006289 0.016230	N -0.391666 0.002888 -0.009392<br>C 0.281176 1.167293 -0.081099<br>C 1.665901 1.199224 -0.106337<br>C 2.369296 0.003691 -0.031763<br>C 1.668015 -1.191955 0.057820<br>C 0.282967 -1.160985 0.052113<br>H -0.323872 2.057835 -0.106621<br>H 2.177088 2.146336 -0.184536<br>H 2.180798 -2.139675 0.115128<br>H -0.320951 -2.052142 0.082516<br>Ge -2.430692 0.001258 0.005032<br>F -2.247549 -1.595775 -0.699967<br>F -2.239606 1.600935 0.701773<br>F -3.266539 0.607259 -1.362238<br>F -3.228856 -0.609744 1.392279

	C	-3.911695	0.002825	0.028991	C	3.874814	0.008037	0.001300
	F	-4.396227	1.057602	0.691417	F	4.371837	1.042357	-0.682255
	F	-4.393260	0.052294	-1.220056	F	4.313843	0.103177	1.262150
	F	-4.391967	-1.103818	0.604162	F	4.375162	-1.114870	-0.520753
	F	1.906487	-0.056677	1.890815	N	0.003335	0.003270	-0.008028
	F	2.057415	1.723588	-0.827337	C	-0.671115	1.170817	0.015773
	F	4.159145	0.012171	0.068799	C	-2.056548	1.204193	-0.007720
	F	2.065316	-1.640019	-0.947568	C	-2.759526	0.007188	-0.059757
	Sn	2.256343	0.010105	0.012170	C	-2.058557	-1.191917	-0.081908
	C	-0.671038	-1.153958	-0.151718	C	-0.673067	-1.162375	-0.056554
	C	-2.056550	-1.194823	-0.117132	H	-0.061540	2.059147	0.052319
	C	-2.061057	1.202543	-0.024633	H	-2.568103	2.154324	0.005656
	C	-0.675473	1.169732	-0.062051	H	-2.571697	-2.140203	-0.127280
4-TFMPy···SnF_4	N	0.000875	0.009780	-0.123356	H	-0.064982	-2.052279	-0.075350
	H	-0.069237	-2.046724	-0.218687	Sn	2.235885	0.000273	0.027984
	H	-2.568746	-2.144119	-0.150223	F	1.986713	-1.917422	-0.035540
	H	-2.576849	2.149647	0.015424	F	1.989906	1.918521	0.087158
	H	-0.077184	2.067360	-0.060049	F	3.122898	-0.054376	1.711848
	C	-2.760155	0.001868	-0.054540	F	3.167445	0.052634	-1.631754
	C	-4.263027	-0.003503	0.028692	C	-4.265503	0.007897	-0.041790
	F	-4.784697	1.095712	-0.523722	F	-4.716733	-0.030833	1.217782
	F	-4.662024	-0.045318	1.306020	F	-4.758914	-1.053855	-0.684365
	F	-4.781266	-1.066318	-0.593592	F	-4.757117	1.108265	-0.617270
